# Misophonia is associated with altered brain activity in the auditory cortex and salience network

**DOI:** 10.1038/s41598-019-44084-8

**Published:** 2019-05-17

**Authors:** Arjan Schröder, Guido van Wingen, Nadine Eijsker, Renée San Giorgi, Nienke C. Vulink, Collin Turbyne, Damiaan Denys

**Affiliations:** 1 Amsterdam University Medical Centers, University of Amsterdam, Department of Psychiatry, Amsterdam, The Netherlands; 2 Amsterdam Neuroscience, Amsterdam, The Netherlands; 3 Amsterdam Brain and Cognition, University of Amsterdam, Amsterdam, The Netherlands; 4 The Netherlands Institute for Neuroscience, an institute of the Royal Netherlands Academy of Arts and Sciences, Amsterdam, The Netherlands

**Keywords:** Aggression, Magnetic resonance imaging

## Abstract

Misophonia is characterized by intense rage and disgust provoked by hearing specific human sounds resulting in social isolation due to avoidance. We exposed patients with symptom provoking audiovisual stimuli to investigate brain activity of emotional responses. 21 patients with misophonia and 23 matched healthy controls were recruited at the psychiatry department of the Amsterdam UMC. Participants were presented with three different conditions, misophonia related cues (video clips with e.g. lip smacking and loud breathing), aversive cues (violent or disgusting clips from movies), and neutral cues (video clips of e.g. someone meditating) during fMRI. Electrocardiography was recorded to determine physiological changes and self-report measures were used to assess emotional changes. Misophonic cues elicited anger, disgust and sadness in patients compared to controls. Emotional changes were associated with increases in heart rate. The neuroimaging data revealed increased activation of the right insula, right anterior cingulate cortex and right superior temporal cortex during viewing of the misophonic video clips compared to neutral clips. Our results demonstrate that audiovisual stimuli trigger anger and physiological arousal in patients with misophonia, associated with activation of the auditory cortex and salience network.

## Introduction

Misophonia is a condition in which individuals experience intense anger and disgust when they are confronted with sounds made by other human beings^1^. In particular, sounds like chewing, lip smacking or breathing may cause intense anger and physical arousal^2,3^. Sufferers usually avoid misophonic situations or endure them with intense discomfort, which leads to profound functional impairment.

In 2013, we were the first to define diagnostic criteria for misophonia as a psychiatric disorder^1^. The paper has been viewed over 155.000 times in the past 5 years. We believe that the symptoms are best explained within the discipline of psychiatry due to [1] pronounced mental suffering of patients, [2] the cognitive (obsessive) and affective nature of the symptoms following conditioning, [3] the behavioral coping strategies such as avoidance, and [4] treatment options which are in the realm of psychiatric discipline. Using the diagnostic criteria, in the past seven years, more than 1000 patients at the Amsterdam UMC (formerly known as Academic Medical Center) were carefully observed and treated.

Because of its novelty, misophonia research is still in its infancy and not readily accepted in the scientific community as a distinct and valid disorder. We believe that detecting neurobiological mechanisms may help receiving misophonia as a genuine disorder and eventually the patients suffering severe symptoms but yet unrecognized by many health care professionals. However, the neurobiological mechanisms of misophonia have rarely been examined.

An initial physiological study, using auditory misophonic triggers, found higher skin conductance responses to auditory stimuli in misophonia patients compared to healthy controls^2^. This increased conductance was thought to reflect an autonomous physical component in the misophonic reaction. More evidence for aberrant physiological reactions was shown in a electroencephalography (EEG) study from our group^4^. In this study we found deviant neuronal activation in the automatic auditory processing system in misophonia patients. This abnormal auditory processing might underlie misophonia symptoms. EEG only enables recording from cortical areas with limited anatomical specificity, whereas the misophonic reaction is likely to additionally engage multiple emotional – limbic – structures^3,5^.

These structures can be examined using functional magnetic resonance imaging (fMRI), which offers better spatial resolution. A recent fMRI study by Kumar *et al*.^6^ found that misophonic trigger sounds elicited increased activation in the anterior insula and abnormal functional connectivity with the ventromedial prefrontal cortex, posteromedial cortex, hippocampus and amygdala, regions involved in emotional processing and regulation. It was suggested that this could reflect abnormal salience attribution to misophonic stimuli. This excellent study contributed to our understanding and acceptance of misophonia. A limitation, however, was that the stimuli presented consisted of sounds only, whereas in regular daily life patients are generally exposed to human sounds with concurrent visual input. Furthermore, some patients also experience similar symptoms following visual triggers^1^.

In the current study, our aim was to identify the brain regions involved during the emotional misophonic reaction. We triggered misophonic symptoms using video clips and measured neural, physiological and behavioral responses. Misophonia patients experience anger and disgust when confronted with misophonic triggers that are perceived as neutral by healthy controls. Hence, we expected that audiovisual misophonic cues would evoke a different neural activity in misophonia patients compared to controls.

We specifically focused on brain regions that are involved in the processing of sounds and aversive stimuli. Because stimuli that are ordinary to most people are experienced as highly salient in misophonia, we expected activation of the salience network, notably the insula and anterior cingulate cortex (ACC). This network segregates important interoceptive and environmental information to ensure proper response selection^7,8^. Since misophonia may be related also to increased vigilance, the capacity to sustain attention over a prolonged time for threat detection^9,10^, we hypothesized enhanced activity in the amygdala^11,12^ and the superior temporal cortex to maintain heightened attention to sounds^13,14^.

## Methods and Materials

### Participants

Twenty-five misophonia patients and twenty-five healthy controls participated in the study, matched for sex, age and educational level. Misophonia patients were all interviewed by three psychiatrists experienced in diagnosing misophonia. Healthy controls underwent screening over the telephone and were interviewed by a psychiatrist (A.S.) to disclose any misophonia symptoms, psychiatric diagnoses, current and previous health issues, medication use, and alcohol or substance use. Exclusion criteria for all participants consisted of major depression, anxiety disorder, bipolar disorder, psychotic disorder, autism spectrum disorder, substance related disorder, hearing loss, MRI contraindications, epilepsy or any structural central nervous system disorder or stroke within the last year. Misophonia patients, who experienced a misophonic reaction to eating sounds and to at least two out of the four sounds used as stimuli for symptom provocation, were included. Two patients had co-morbid AD(H)D, one had a diagnosis of borderline personality disorder in the past. Methylphenidate (30 mg daily) was used by one patient. Four patients and two controls were excluded from the final analyses because of various reasons (see Table 1). The study was approved by the Medical Ethics Trial Committee (METC) of the Academic Medical Center (AMC) Amsterdam and written informed consent was obtained from all participants. All methods were performed in accordance with relevant guidelines and regulations.

**Table 1:** Clinical and demographic characteristics.

	Patients	Controls	Statistical analysis	
	(N = 21^a^)	(N = 23^a^)	(df = 42)	*p*-value
Male (N, %)	6 (28%)	6 (26%)	*χ²* = 0.034^e^	0.853
Age (Mean, SD)	33.1 (9.9)	33.4 (9.8)	*t* = −0.114	0.910
Age of onset (Mean, SD)	12.2 (3.2)			
Educational level (mean)^b^	5	5		
**Measures** ^**c**, **d**^				
A-MISO-S (Mean, SD)	15.1 (2.8)			
SCL-90 (Mean, SD)	148.8 (46.0)	103.6 (14.1)	*U* = 70.5^f^	<0.001^g^
HAM-A Mean, SD)	12.6 (8.7)	2.6 (3.6)	*U* = 70.5^f^	<0.001^g^
HAM-D (Mean, SD)	9.3 (6.3)	1.7 (2.5)	*U* = 47.0^f^	<0.001^g^
**BPAQ** (**Mean**, **SD**)				
Physical Aggression	19.6 (6.1)	16.4 (3.4)	*U* = 147.0	0.026
Verbal Aggression	12.1 (2.6)	12.0 (2.6)	*U* = 240.0	0.972
Anger	20.7 (5.8)	13.4 (3.9)	*U* = 73.5	<0.001^g^
Hate	20.7 (8.1)	14.2 (4.0)	*U* = 129.0	0.008
Total Anger	73.1 (18.6)	56.0 (9.6)	*U* = 106.5	0.001^g^

^a^Excluded: 1 patient and 2 controls because of buzzing sound in audiosystem; 1 patient because of absence of sound; 2 patients because MRI data were unusable due to recording errors.

^b^Educational level was assessed according to the ISCED system, ranging from 0 (no finished education) to 8 (finished university training). Median: ISCED level 6.

^c^A-MISO-S = Amsterdam Misophonia Scale; SCL-90 = Symptom Checklist; HAM-A = Hamilton Anxiety Rating Scale; HAM-D = Hamilton Depression Rating Scale; BPAQ = Bush Perry Aggression Questionnaire.

^d^Missing data: SCL90: 1 patient, 1 control; HAM-A/HAM-D: 2 controls.

^e^*χ²;* Chi-square test (df = 1).

^f^(df = 40).

^g^Significant with Bonferroni correction *p* < 0.05/8.

### Questionnaires

During screening, misophonia symptom severity in misophonia patients was assessed with the Amsterdam Misophonia Scale (A-MISO-S)^1^. General mental and physical dysfunctioning was measured with the Dutch version of the Symptom Checklist (SCL-90)^15,16^. The total score provides an index for psychoneuroticism. Anxiety and depressive symptoms were assessed with the Hamilton Anxiety Rating Scale (HAM-A) and Hamilton Depression Rating Scale (HAM-D), respectively^17,18^. Aggressive personality style was assessed with the Dutch version of the Buss-Perry Aggression Questionnaire (BPAQ)^19,20^. It consists of four categories - physical aggression (PA), verbal aggression (VA), anger (A) and hostility (H) - and a total score, which is considered a general index of trait aggression. After scanning, visual analogue scale (VAS) ratings were used to score how much anger, anxiety, happiness, sadness, and disgust each clip evoked for the participant personally. Due to recording issues, VAS scores were available for 39 subjects (18 patients, 21 controls). A short form (32 items) of the Dutch version of the Profile of Mood States (POMS-SF)^21,22^ was used to rate mood and level of arousal, both before and after scanning. The 32 items were divided into five categories: four negative moods states (“depressed”, “angry”, “fatigued”, “tension“) and one positive (“vigorous”), with “angry” subscore as the key emotion of interest. Total mood disturbance score was calculated by adding up the four negative mood scores and subtracting the positive one.

### Physiological measurements

ECG was recorded during scanning to monitor baseline heart rate and changes in heart rate during symptom provocation. Inter-beat-intervals (IBIs) were calculated for each clip. Segments with artifacts were excluded and clips with more than 10% missing IBI data points were removed. Valid ECG recordings were available for 14 patients (215 out of 224 video clips) and 19 controls (290 out of 304 video clips).

### fMRI paradigm

In the symptom provocation paradigm, neutral, misophonic, and aversive audiovisual stimuli were shown to the participants while inside the MRI scanner. We included a generally aversive condition to assess whether different responses in patients would generalize to non-misophonia specific cues and as confirmation that activity could also be triggered in controls. The clips had been produced and tested in a previous pilot study in our lab with two patients and six controls, who did not participate in the current study. Neutral clips were experienced as neutral by both patients and controls. Aversive clips evoked aversive reactions in both groups, while misophonic clips were experienced as being aversive solely by patients.

Neutral clips displayed a male actor performing soundless activities, which included meditating, reading a book, writing in a notebook, and handling a tablet computer. Misophonic clips displayed another male actor producing typical misophonic trigger sounds, i.e. eating a carrot, eating a grapefruit, typing and heavy breathing. Aversive clips displayed segments of very violent or loathsome scenes from several commercial films (see Supplement 1). Auditory levels of the video clips were not controlled for.

Stimuli were presented in a blocked design. The three conditions and a fixation block each lasted 25 seconds with a 2 seconds inter-stimulus interval. The order of conditions was fixed in a pseudorandom order and the order of the video clips within each condition was randomized. The symptom provocation paradigm consisted of four blocks per condition.

### Image acquisition and pre-processing

Anatomical and functional images were acquired using a Philips Ingenia 3.0 T MRI system (Philips Medical Systems, Best, the Netherlands) with a SENSE 32 elements head coil. An anatomical T1-weighted image (3D MP-RAGE) was acquired for normalization purposes [voxel size = 1 mm^3^, TR/TE = 7000/3.2 ms, matrix = 256 × 256, field of view (FOV) = 256 × 240 mm, 180 sagittal slices]. Functional images were acquired using T2*-weighted Echo-Planar Imaging (EPI) [TR/TE = 2000/27 ms, matrix = 80 × 80, in-plane resolution = 3 × 3 mm, slice thickness = 3 mm, slice gap = 0.3 mm, 37 axial slices].

Imaging data were analyzed with SPM8 (Wellcome Trust Centre for Neuroimaging, London). During pre-processing, images were realigned to correct for motion-related artifacts and slice-timing correction was applied for differences in acquisition time. Images were then coregistered with the anatomical image (MP-RAGE) and normalized to the Montreal Neurological Institute (MNI) space template using segmentation of the anatomical scan. Data were resliced with a 2 × 2 × 2 mm resolution and spatially smoothed with an 8 mm full width at half maximum Gaussian kernel.

### Data analysis

#### Questionnaires

Statistical analysis was performed with SPSS, version 20 (IBM, 2011), using non-parametric Mann-Whitney *U*-tests with Bonferroni corrections for multiple comparisons. SCL90, HAM-A, HAM-D and BPAQ scores were compared between groups. For the VAS analysis, a mean VAS score for each emotion for every condition was calculated. These emotion scores were compared between groups for each condition. To examine mood changes due to the paradigm, we calculated change scores on the POMS-SF subscales and total score. Possible differences in change scores between the groups were investigated.

#### Physiology

The mean IBI for each clip was calculated and subsequently averaged for all clips per condition. Group x condition interactions and group effects were assessed using a general linear model (GLM). Paired-samples *t*-tests were carried out for the mean IBIs between the conditions within the groups (Bonferroni correction *p* < 0.05/3).

#### Imaging

For the first level model estimation, the three conditions (misophonic, aversive, and neutral) were modeled as boxcar regressors and convolved with the canonical hemodynamic response function (HRF). Realignment parameters were adopted as additional regressors to control for variance due to head movements. A high-pass filter (128 seconds) was applied to remove slow signal drifts. Temporal autocorrelation was modeled using an autoregressive AR(1) process. Contrast maps for all three conditions were obtained for each participant and entered in second level analyses. To test group differences in responses to the misophonic condition, one interaction analysis compared responses between the misophonic and neutral conditions between groups. To test whether the groups also differed in responses to generally aversive stimuli, another interaction analysis compared responses between the aversive and neutral conditions between groups. To test whether potential differences were specific for misophonic stimuli, an additional interaction analysis compared responses between the misophonic and aversive conditions between groups. Voxel-wise statistical tests were family-wise-error (FWE) rate corrected for multiple comparisons (p < 0.05) across the whole brain at the cluster level, using cluster-forming threshold of p < 0.001, or at the peak level for the preselected four regions of interest (ROI) analyses, using a small volume correction (p < 0.05). ROIs for the insula, ACC, amygdala and superior temporal cortex were defined using the Anatomical Automatic Labeling (AAL) toolbox^23^. ROIs were bilateral. Statistical corrections were done per ROI.

To perform additional correlational analyses between neuroimaging, behavioral, and physiological data, we extracted activity estimates from the peak voxel of ROIs that showed between-condition differences of the patients. We correlated those values to clinical scores (misophonia severity, aggressive personality style, VAS scores and POMS-SF anger change scores) and to physiological data (baseline heart rate and heart rate increase during the misophonic condition).

## Results

### Group characteristics and behavioral data

Clinical and demographic characteristics are provided in Table 1. Patients showed overall more psychiatric symptoms (SCL90; p < 0.001), anxiety symptoms (HAM-A; p < 0.001) and depression symptoms (HAM-D; p < 0.001) than controls. Moreover, BPAQ scores showed that patients reported more anger (p < 0.001) and total anger (p = 0.001).

**Figure 1:**
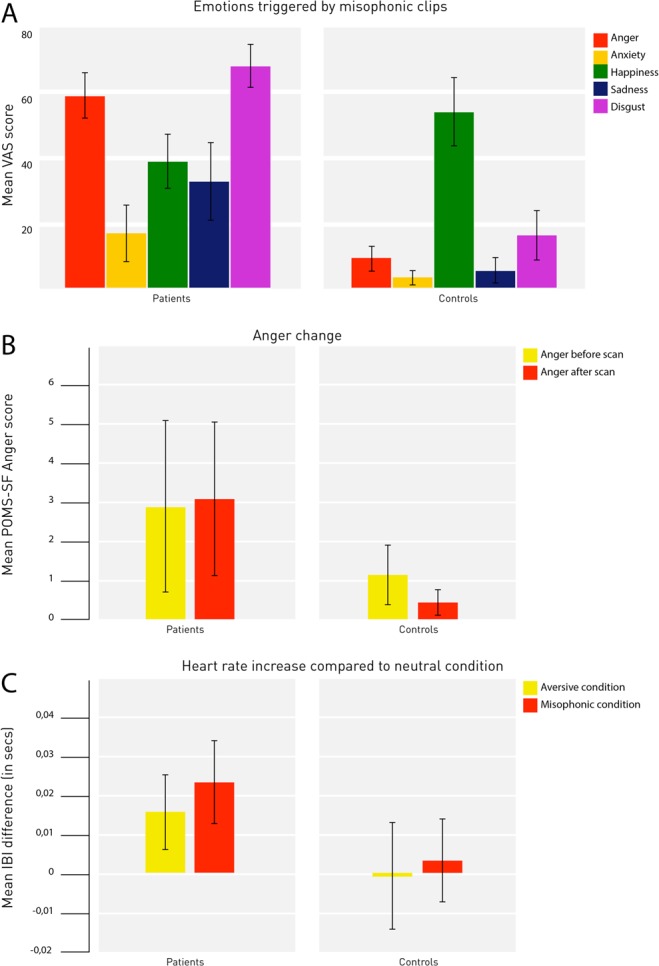
(**A**) Visual analogue scale (VAS) ratings (range from 0–100) were used to score how much anger, anxiety, happiness, sadness, and disgust each clip evoked for the participants personally. Misophonic clips provoked more anger (p < 0.001), disgust (p < 0.001) and sadness (p < 0.001) in patients compared to controls. (**B**) Change in anger sub scores on the POMS-SF. Graphs show the two mean anger sub scores filled out before and after the paradigm. Misophonic patients had higher anger scores before and after. The difference in anger change between the two groups was significant (p < 0.048). (**C**) Heart rate was recorded as the interval between two heartbeats, the inter-beat-interval (IBI). Smaller IBI means faster heart rate, reflecting increased physiological arousal. Patients showed larger differences between the mean IBIs for the misophonic and neutral condition (p < 0.001) and between the aversive condition and neutral condition (p = 0.007), i.e. more physiological arousal during the misophonic and aversive condition. No differences were found for controls.

Analyses of emotions (VAS-scores), which were triggered by the different conditions showed that misophonic clips provoked more anger (p < 0.001), disgust (p < 0.001) and sadness (p < 0.001) in patients than in controls (Table 2; Fig. 1). Importantly, no differences between groups were found in triggered emotions for generally aversive clips (Table 2).

**Table 2:** Triggered emotions (VAS scores), POMS-SF change scores and physiological measurements.

			Patients	Controls	Statistical analysis		
			(Mean, SD)	(Mean, SD)	Mann-Whitney *U* test		*p*-value
Triggered emotions	Condition		(N = 21)	(N = 23)			
VAS scores	Misophonic	Anger	59.14 (14.99)	8.96 (9.04)	0.000		<0.001^a^
		Anxiety	16.84 (18.47)	3.21 (5.24)	107.5		0.020
		Happiness	39.06 (17.76)	54.13 (24.38)	107.0		0.020
		Sadness	32.79 (25.51)	5.38 (9.45)	67.5		<0.001^a^
		Disgust	68.31 (14.32)	16.33 (17.65)	6.0		<0.001^a^
	Aversive	Anger	57.06 (21.44)	40.76 (23.54)	114.5		0.035
		Anxiety	39.88 (26.49)	33.06 (24.94)	165.5		0.512
		Happiness	31.61 (23.07)	33.19 (25.56)	188.0		0.989
		Sadness	52.58 25.49)	34.14 (21.44)	104.5		0.016
		Disgust	70.75 (17.69)	61.32 (28.67)	164.5		0.494
	Neutral	Anger	11.03 (19.65)	4.45 (5.91)	176.0		0.728
		Anxiety	11.90 (18.71)	2.69 (4.77)	156.5		0.364
		Happiness	59.75 (25.70)	53.60(24.02)	166.5		0.530
		Sadness	11.75 (16.76)	6.79 (14.26)	168.0		0.568
		Disgust	11.13 (18.16)	3.70 (5.60)	172.5		0.646
**Mood change after paradigm**							
**POMS-SF change scores**							
		Anger	0.19 (3.41)	−0.70 (1.77)	164.5		0.048^b^
		Depression	0.48 (3.61)	0.44 (1.15)	203.0		0.310
		Fatigue	1.24 (2.74)	0.61 (1.67)	193.0		0.246
		Vigor	−2.00 (3.58)	−1.26 (4.27)	201.5		0.343
		Tension	−2.62 (3.40)	−2.22 (2.84)	228.5		0.754
		Total Mood Disturbance	1.81 (12.16)	−0.57 (6.65)	189.5		0.221
**Physiological measurements**							
**Inter beat interval (IBI) (Mean, SD)**	**Condition**		**(N = 14)**	**(N = 19)**	**Paired** ***t*** **-test**		
	Misophonic		0.832 (0.089)	0.985 (0.138)	Misophonic vs neutral:		
					Patients	−4.385^c^	<0.001
					Controls	0.632^d^	0.535
	Aversive		0.839 (0.082)	0.989 (0.141)			
					Aversive vs neutral:		
	Neutral		0.854 (0.082)	0.988 (0.134)	Patients	3.229^c^	0.007
					Controls	−0.088^d^	0.931

^a^Significant with Bonferroni correction *p* < 0.05/15.

^b^Significance level *p* = 0.05.

^c^df = 13.

^d^df = 18.

Analysis of the POMS-SF scores obtained before and after the experiment showed that patients reported a significantly larger increase in anger than controls (p = 0.048) (Table 2). Follow-up tests revealed that this was due to a near-trend decrease in controls (p = 0.072), whereas anger levels of patients did not change (p = 0.574) (Fig. 1).

### Physiological data

To investigate whether there was an effect on heart rate, we performed a repeated measures ANOVA. This revealed a significant group x condition interaction effect (F(2,62) = 3.722; p = 0.030) and a main effect of condition (F(2,62) = 5.967; p = 0.004). Furthermore, a main effect of group showed that patients had significantly smaller IBIs than controls across conditions (F (1,31) = 12.275, p = 0.001). Follow-up paired-samples *t*-tests showed smaller IBIs in patients during both the misophonic (t = −4.385, df = 13, p = 0.001) and the aversive condition (t = 3.229, df = 13, p = 0.007) compared to the neutral condition, reflecting an increased heart rate (Table 2; Fig. 1), whereas IBIs for controls were similar over the three conditions.

### Imaging data

There was a significant main effect of condition showing large clusters of activation around occipital, parietal and superior temporal cortices, for both the misophonic and the aversive condition compared to the placid neutral clips (Supplement 2), reflecting audiovisual activation during these video clips. Furthermore, a main effect of group was observed, with reduced activity in the right inferior temporal gyrus in patients compared to controls (p_FWE_ = 0.001) (Table 3). In the whole brain analysis no additional results were found.

**Table 3:** Brain areas that show increased activation.

Test	Region	Side	Cluster size		MNI		Z	*p*_FWE-SVC_
				*x*	*y*	*z*		
*Main effect of group controls >patients*	Inferior temporal gyrus	R	890	44	−62	−12	4.29	0.001
	Fusiform gyrus	R		34	−78	−14	4.36	
**patients >controls misophonic condition >neutral condition**								
	Insula	R	45	32	12	−14	3.75	0.030
	Superior temporal cortex	R	246	60	−26	6	3.77	0.035
	Anterior cingulate cortex	R	237	4	44	16	3.48	0.046

**Figure 2:**
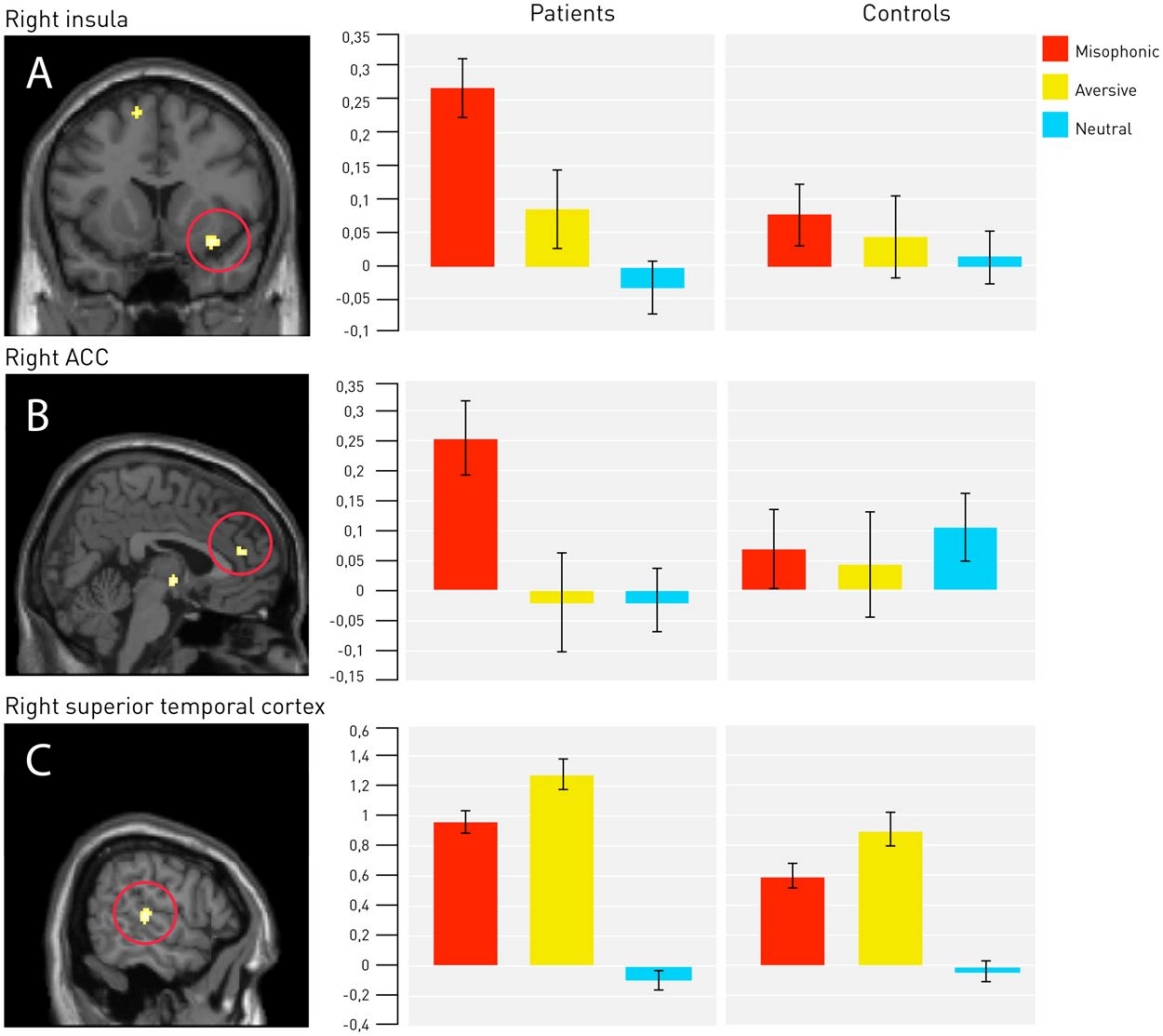
Statistical maps showing increased activation in patients during the misophonic condition in three regions of interest: (**A**) right insula (pSVC = 0.030), (**B**) right ACC (pSVC = 0.046), and (**C**) right superior temporal cortex (pSVC = 0.035).

To explore the primary misophonic reaction, we examined group differences in the response to the misophonic condition compared to the neutral condition. This analysis revealed a group x condition interaction effect, with increased activity in patients in the right insula (p_SVC_ = 0.030), right ACC (p_SVC_ = 0.046), and right superior temporal cortex (p_SVC_ = 0.035) (Table 3; Fig. 2). No group differences were found when comparing the aversive with the neutral condition. Even though group differences were only observed in the misophonic condition, the group x condition interaction between the misophonic and aversive conditions showed no significant effects.

Peak voxel activity, extracted from the three significant clusters, was not correlated with clinical scores or heart rate measurements.

## Discussion

Our results show that specific audiovisual cues may elicit [1] anger, disgust, and sadness in patients with misophonia, and that they are accompanied by [2] increased physiological arousal, and [3] increased activity in the right insula, right ACC, and right superior temporal cortex. These different brain responses were only observed in the misophonic condition. Brain responses to generally aversive stimuli were not significantly different between patients and controls. The absence of differences between the misophonic and aversive conditions suggests that this could be due to a subthreshold and non-significant increase in response in patients in the aversive condition. As such it may reflect exaggerated responses to aversive stimuli in misophonia patients in general.

The recruitment of the insula, ACC and superior temporal cortex suggests that salience attribution to misophonic cues may underlie the symptoms observed in misophonia. First, misophonic cues initially provoke physiological arousal and aversive emotions. Second, repeated exposure to the same cues will amplify the salience network activity. The mechanism reflects a conditioned response in which the initially neutral stimulus is increasingly associated with intensified aversive emotions^24,25^, and augmented by increased vigilance.

Even though patients suffered more general psychiatric symptoms and aggressive personality styles than controls, they were not angrier before being exposed to the misophonic video clips. Following the experiment, patients were significantly angrier than controls, reflecting their main symptoms. This selective response is important. Since other emotions were not observed following the paradigm, it is unlikely that group differences are related to changes in anxiety or depressive symptoms. The analysis of VAS scores showed that misophonic video clips, but not the aversive video clips, elicited more anger, disgust and sadness in patients compared to controls. Since ratings for aversive video clips were similar for both groups, we assume that anger, disgust and sadness triggered by misophonic stimuli are specific for misophonia patients. We conjecture that the sadness elicited in patients could reflect despair, which accompanies the intense anger and disgust when repeatedly confronted with misophonic cues.

The increased heart rate during the misophonic and aversive conditions in patients suggests apparent autonomic arousal evoked by misophonic and aversive stimuli, and are in line with Edelstein *et al*.^2^ who suggested that patients with misophonia experience extreme aversive reactions in general.

Increased BOLD responses in the right ACC and right insula in patients reflect activation of the salience network. The ACC and insula are key nodes within this network^7^, of which the core function is detection and selection of emotionally salient information^8^. Quick discrimination between relevant and irrelevant information prepares for adequate behavioral responses^26^. ACC and insular activity has been linked also to increased cardiovascular arousal^27^. Misophonic cues are considered highly salient by misophonia patients, driven by heightened autonomic responses.

Interestingly, insular activity has been linked also to disgust^28,29^. This is underlined by the increased subjective averseness-ratings by patients for the misophonic clips. Patients consider misophonic triggers usually as morally unacceptable^1^. Previous research has suggested that insular and ACC activity is implicated in moral assessment of stimuli^30,31^ which mediates attentional processes^8^. It is possible that patients could have perceived these stimuli as a personal harassment, thus triggering subsequent anger. A previous study comparing various anger provocation methods showed that only methods that included personal contact, i.e. an interview or harassment of subjects, increased both self-report levels of anger and physiological reactivity^32^.

Patients also showed hyperactivity of the right superior temporal cortex. This region plays a central role in selective auditory attention, especially in processing emotionally salient sounds^26,33^ explaining why misophonic cues increase auditory attention in patients. Sensitization of the auditory cortex may cause an increased response to a specific stimulus^34^, and on its turn amplify the patient’s emotional salience system, labeling misophonic sounds as being emotionally relevant. Importantly, even though audio levels of the clips of the three conditions were not controlled for, audio levels were the same in both groups during the paradigm, which strengthens these findings.

The absence of significant differences in amygdala activity was unexpected. A possible explanation is that the amygdala is specifically implicated in the processing of fear^35^. Reviews reveal that the amygdala is activated in sixty percent of studies examining fear^36^ and fewer than twenty percent of studies examining disgust, anger, happiness, or sadness^35^. The absence of activity in the amygdala is consistent with the absence of increased fear levels on the behavioral data. This finding underscores that anxiety is not a primary emotion in misophonia^1^, although it could still develop over time as anticipatory anxiety.

Our imaging results are in line with the study by Kumar *et al*.^6^, which reported as well that misophonic sounds were associated with activation of the anterior insula and ACC, and heightened heart rate. They were the first to postulate that misophonia is mediated by abnormalities in the salience network. The results of this independent study confirm their hypothesis.

Unexpectedly, controls showed a large activated cluster in the right inferior temporal gyrus and fusiform face area. These regions are involved in recognition of human bodies, notably faces^37,38^. We do not understand how this finding relates to misophonia. Our previous EEG study suggested a defect in the processing of human sounds in misophonia patients^4^, but our fMRI results indicate that visual processing of human images may be implicated as well. It should be noted that in our study we only employed human stimuli because we believe these are the core triggers for misophonia. However, basically a myriad of sounds – human or non-human - could become salient and connected to an aversive response depending on context.

Noteworthy, the average duration of misophonia symptoms in our patient group was 21 years. It is plausible that longer duration, with repetitive exposure to misophonic triggers, could therefore be a factor for increased reactions^39^.

Our study has several limitations. First, the presentation of stimuli in a controlled laboratory setting lacks ecological validity. Gradient noise interferes with auditory stimulation^26,40^ and can induce stress and annoyance^26,41^. Second, each misophonia patient has an individual pattern of misophonic trigger sounds that they react to. Therefore, the applied trigger sounds in the current study may not have evoked the maximal misophonic response in all the patients. However, to tailor all triggers for all participants was out of reach in this study. Third, there may have been a selection bias of participants. Patients with the most extreme misophonic reactions could have refused participation for fear that being in the scanner itself may be too high of a burden to endure. Fourth, our results do not differentiate between misophonic and aversive triggers. Therefore, it cannot be excluded that misophonic symptoms are related to increased averseness levels.

In conclusion, we explored the neural correlates of misophonia with audiovisual symptom provocation. Based on our findings, we posit that misophonia involves a conditioned response with anger and physical arousal elicited by human audiovisual triggers. The symptoms are mediated by enhanced reactivity of the salience network in combination with hypervigilance, reflected by sensitization of the auditory cortex.

## Supplementary information

SUPPLEMENTARY INFORMATION
