# Publisher Correction: Misophonia is associated with altered brain activity in the auditory cortex and salience network

**DOI:** 10.1038/s41598-020-59862-y

**Published:** 2020-02-28

**Authors:** Arjan Schröder, Guido van Wingen, Nadine Eijsker, Renée San Giorgi, Nienke C. Vulink, Collin Turbyne, Damiaan Denys

**Affiliations:** 1 Amsterdam University Medical Centers, University of Amsterdam, Department of Psychiatry, Amsterdam, The Netherlands; 2 Amsterdam Neuroscience, Amsterdam, The Netherlands; 3 Amsterdam Brain and Cognition, University of Amsterdam, Amsterdam, The Netherlands; 4 The Netherlands Institute for Neuroscience, an institute of the Royal Netherlands Academy of Arts and Sciences, Amsterdam, The Netherlands

Correction to: *Scientific Reports* 10.1038/s41598-019-44084-8, published online 17 May 2019

In the original version of this Article, the author Guido van Wingen was incorrectly indexed. This error has now been corrected.

