# Supplementary material for: Misophonia is associated with altered brain activity in the auditory cortex and salience network
Source: Sci Rep. 2019 May 17;9:7542. doi: 10.1038/s41598-019-44084-8 (PMC6525165; doi:10.1038/s41598-019-44084-8)
Supplement: Supplementary file 1 — SUPPLEMENTARY INFORMATION [file 41598_2019_44084_MOESM1_ESM.docx]

*Misophonia is associated with altered brain activity in the auditory cortex and salience network*

Arjan Schröder, M.D., Guido van Wingen, Nadine Eijsker, Renée San Giorgi, Collin Turbyne, Nienke C. Vulink, M.D., Ph.D., Damiaan Denys, M.D., Ph.D.

**SUPPLEMENTARY INFORMATION**

**Supplement. Main effects of condition**
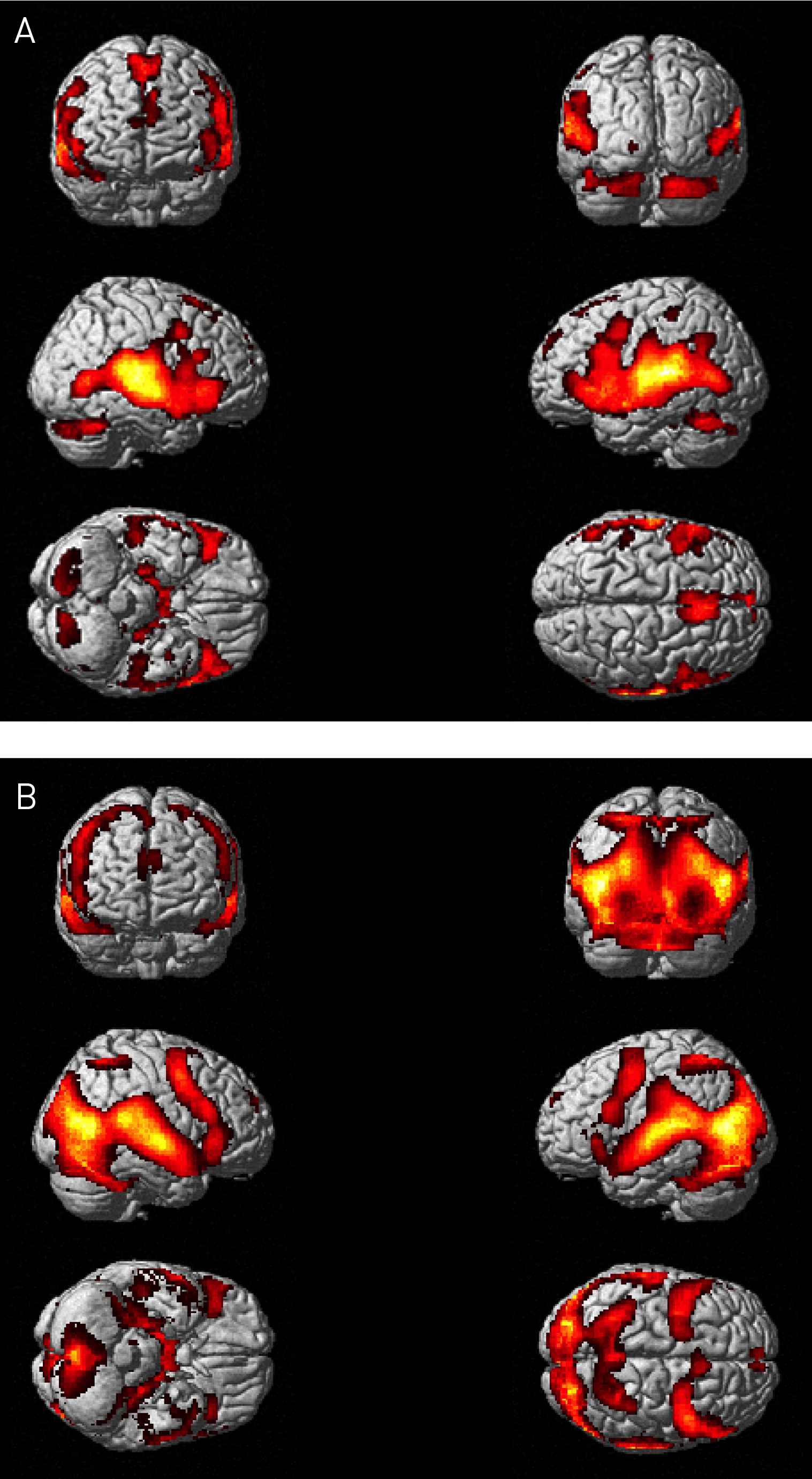


Activation maps showing main effects of condition, for (A) misophonic compared to neutral condition and (B) aversive compared to neutral condition. Both maps show large activated clusters centered around occipital, parietal and superior temporal cortices. This activity reflects the audiovisual stimulation of the misophonic video clips. The neutral clips consisted of more placid scenes with less sound or visual movement.
